# Clinical, Radiological, and Pathological Features of Intraosseous Hibernoma: A Systematic Review of Case Reports and Case Series

**DOI:** 10.3390/curroncol32100535

**Published:** 2025-09-24

**Authors:** Jawad Albashri, Ahmed Albashri, Muhannad Alhamrani, Abdulrahman Hassan, Hisham Shamah, Rayan Alhefzi, Najim Z. Alshahrani, Mohammed R. Algethami, Louis-Romée Le Nail, Ramy Samargandi

**Affiliations:** 1 College of Medicine, University of Jeddah, Jeddah 23218, Saudi Arabia; 2 Department of Family and Community Medicine, College of Medicine, University of Jeddah, Jeddah 23218, Saudi Arabia; 3 Orthopaedic Surgery Department, Centre Hospitalier Régional Universitaire (CHRU) de Tours, 37170 Tours, France; 4 Orthopaedic Surgery Department, College of Medicine, University of Jeddah, Jeddah 23218, Saudi Arabia

**Keywords:** intraosseous hibernoma, brown adipose tissue, rare bone tumors, metastasis mimicker, PET imaging, MRI, histopathology, systematic review

## Abstract

Intraosseous hibernoma (IOH) is a rare benign tumor composed of brown adipose tissue within the bone, frequently mimicking metastatic lesions and leading to diagnostic challenges. This systematic review aimed to consolidate and analyze all published IOH cases to improve recognition and inform management. A comprehensive literature search was conducted in PubMed, Web of Science, Scopus, Google Scholar, and the Cochrane Library from database inception to March 2025. Studies were eligible for inclusion if they reported histopathologically confirmed cases of intraosseous hibernoma (IOH) in human patients. A total of 62 cases from 30 studies were included. The mean age was 59.2 years, with a female predominance. Lesions were most frequently located in the pelvis and spine and were typically identified incidentally during cancer staging or imaging performed for unrelated indications. Imaging often revealed sclerotic patterns on computed tomography (CT), hyperintense signals on magnetic resonance imaging (MRI) T2-weighted and short tau inversion recovery (STIR) sequences, and mild to moderate uptake on ^18^F-fluorodeoxyglucose positron emission tomography/computed tomography (^18^F-FDG PET/CT). Immunohistochemistry consistently showed S100 protein positivity. Most patients underwent biopsy and were managed conservatively, with no cases of malignant transformation reported. IOH is a benign entity with distinctive radiologic and immunohistochemical features that may mimic malignancy. Awareness of its presentation can reduce misdiagnosis and unnecessary interventions, supporting biopsy-based confirmation and conservative management in most cases.

## Simple Summary

Intraosseous hibernoma (IOH) is a rare benign tumor composed of brown fat within the bone. Its unusual imaging features often mimic metastatic disease or primary bone tumors, leading to unnecessary investigations or treatments. This systematic review synthesized all published case reports and case series to clarify its clinical, radiological, histopathological, and management characteristics. We identified 62 confirmed cases from 30 publications. Most lesions were found incidentally, commonly during cancer staging, with the pelvis and spine as the most frequent sites. Radiologically, IOH typically appears as a solitary sclerotic lesion with variable PET/CT uptake, while histology reveals brown adipose tissue with strong S100 positivity. Nearly all patients were managed conservatively following biopsy. Recognizing IOH as a benign mimicker is essential to avoid misdiagnosis and overtreatment, thereby improving patient care.

## 1. Introduction

Hibernomas are rare, benign tumors originating from brown adipose tissue (BAT), most frequently identified in adults [1,2]. It was initially identified by Merkel in 1906, who described it as a “pseudolipoma”, and later named hibernoma by Gery in 1914 due to its histological similarity to the brown adipose tissue found in hibernating species [3]. Brown adipose tissue, which is highly vascularized and rich in mitochondria, is essential for thermogenesis in infancy and gradually regresses with age, although remnants may persist into adulthood and give rise to hibernomas under certain conditions [2,4]. Embryologically, brown adipose tissue derives from mesenchymal stem cells, which also differentiate into skeletal muscle [1,5]. The persistence of BAT into adulthood has important clinical implications, as its activity has been associated with energy metabolism, obesity, and overall metabolic health [4,5].

Soft tissue hibernomas are rare, accounting for less than 1% of benign adipocytic tumors. They usually present as slow-growing, painless masses in the thigh, upper trunk, or neck, typically affecting adults aged 30–40 years with no clear gender predominance [1,2,6]. Intraosseous hibernoma (IOH) is an extremely rare variant that occurs within bone. The first IOH case was described by Thorns et al. in 2008 [7], and only a limited number of cases have since been reported in the literature [6,8]. IOH may radiologically resemble a wide spectrum of bone lesions, ranging from metastases to benign and malignant primary tumors. This overlap frequently leads to diagnostic confusion and the risk of overtreatment [9,10,11]. Radiologically, IOH often presents as a well-defined sclerotic or lytic lesion with variable uptake on ^18^F-fluorodeoxyglucose positron emission tomography/computed tomography (^18^F-FDG PET/CT), which can be misinterpreted, particularly in oncologic settings [12,13,14].

Histopathological examination reveals the presence of brown adipose tissue with characteristic cytological features, including multivacuolated cells with granular eosinophilic cytoplasm and central nuclei. Immunohistochemical staining for S-100 protein is typically positive, aiding in distinguishing hibernoma from other adipocytic or histiocytic tumors [2,8]. A comprehensive evaluation of the clinical, radiological, and pathological features of IOH is necessary to improve diagnostic accuracy and guide management.

This systematic review aims to synthesize all reported cases of intraosseous hibernoma to date, with a focus on clinical presentation, imaging features, histopathology, and management. By consolidating existing evidence, the review seeks to improve diagnostic recognition and guide appropriate clinical decision-making.

## 2. Materials and Methods

### 2.1. Search Strategy and Study Selection

A systematic review of case reports and case series was conducted in accordance with the Preferred Reporting Items for Systematic Reviews and Meta-Analyses (PRISMA) 2020 guidelines [15]. The study protocol was prospectively registered with the International Prospective Register of Systematic Reviews (PROSPERO; registration number CRD42025637236). A comprehensive literature search was performed across the following electronic databases: PubMed, Scopus, Web of Science, Cochrane Library, and Google Scholar, from their inception through March 2025. The search strategy combined terms such as “hibernoma”, “intraosseous”, “intra-osseous”, and “bone”, using Boolean operators (AND, OR) tailored to each database’s syntax.

Two reviewers independently screened titles and abstracts for relevance. Full-text articles were retrieved for all studies meeting initial inclusion criteria. Any disagreements were resolved through discussion or third-party adjudication. Reference lists of included studies were also reviewed to identify any additional eligible publications.

Studies were eligible if they reported histopathologically confirmed IOH in human patients and provided relevant clinical, radiological, or pathological information. Only original research articles (case reports, case series, observational studies, or retrospective analyses) were included. Studies were excluded if they were review articles, editorials, animal studies, not published in English, or if they lacked sufficient diagnostic detail or described hibernomas located outside of bone.

### 2.2. Data Extraction and Quality Assessment

A standardized data extraction form was developed in Microsoft Excel to systematically collect information from each included study. The form encompassed comprehensive variables, including publication details (author, year, journal, country); patient demographics (age, sex); clinical presentation (symptoms, duration, reason for investigation); anatomical location; radiological characteristics across multiple imaging modalities such as computed tomography (CT), magnetic resonance imaging (MRI), ^18^F-FDG PET/CT, and bone scintigraphy/single-photon emission computed tomography (SPECT); immunohistochemical profile; molecular/genetic data; management approaches; and outcomes.

For radiological assessment, data were extracted on lesion appearance (lytic, sclerotic, occult, or mixed), MRI signal characteristics, and standardized uptake values (SUV) on ^18^F-FDG PET/CT when available. For histopathological evaluation, immunohistochemical markers such as S100 (S100 calcium-binding protein), adipophilin (adipophilin protein), FABP4/AP2 (fatty acid–binding protein 4/adipocyte protein 2), CD68 (cluster of differentiation 68), Ki-67 (proliferation index), and others were recorded.

The methodological quality of the included case reports and case series was assessed using the tool developed by Murad et al. [16] for evaluating such study designs in systematic reviews. Each study was appraised across four domains: selection, ascertainment, causality, and reporting. Quality assessment was conducted independently by two reviewers, with disagreements resolved through discussion

### 2.3. Data Synthesis and Statistical Analysis

Due to the nature of the included studies (case reports and case series) and the heterogeneity of reporting, a narrative synthesis approach was used rather than meta-analysis. Findings were organized according to the clinical, radiological, and pathological features of IOH. Descriptive statistics were applied to continuous variables (means, standard deviations, medians, ranges) and categorical variables (frequencies, percentages).

Correlations between clinical presentation, anatomical location, radiological findings, and pathological features were examined using both univariate and multivariate analyses. For categorical variables, chi-square or Fisher’s exact tests were applied as appropriate, while Student’s *t*-test or the Mann–Whitney U test was used for continuous variables according to data distribution. Odds ratios (OR) with 95% confidence intervals (CI) were calculated to quantify associations, including sex with anatomical location, sex with pain, sex with radiological appearance, and location with radiological appearance. Multivariate analyses were adjusted for potentially confounding variables including age, lesion size, and presence of pain (except when pain was the dependent variable).

All statistical analyses were performed using R version 4.4.2 with a significance level set at *p*-value less than 0.05. Graphics and visualization of geographic distribution, symptom-radiology correlation, and anatomical-symptom distribution were generated using Python with matplotlib and seaborn libraries.

## 3. Results

### 3.1. Demographics and Characteristics of Intraosseous Hibernoma Cases

**Figure 1:**
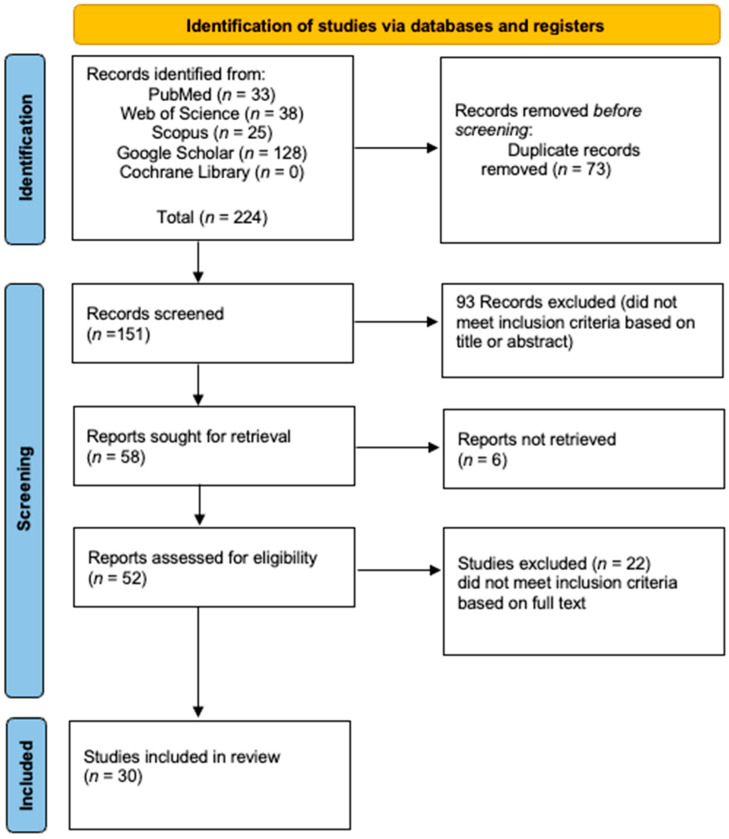
The PRISMA flowchart.

A total of 62 cases of IOH were identified from 30 eligible case reports and case series published between 2008 and 2025 [6,7,8,9,10,11,12,13,17,18,19,20,21,22,23,24,25,26,27,28,29,30,31,32,33,34,35,36,37,38], as illustrated in the PRISMA flowchart (Figure 1). Given the extreme rarity of IOH, the vast majority of these were single-patient reports [6,7,9,10,11,12,17,18,19,20,21,22,23,25,26,27,28,29,30,31,32,33,34,35,36,38], while only a few small series have been published [8,13,24,37], with sample sizes ranging from 1 to 18 cases. Collectively, the studies originated primarily from the United States, Europe, and Asia, reflecting global documentation of this rare entity. The mean age at diagnosis was 59.2 ± 13.13 years, with a reported age range of 7 to 85 years. A female predominance was observed, with 40 female patients (64.52%) compared to 22 males (35.48%). Regarding clinical presentation, 38 patients (61.29%) were identified incidentally—26 (41.94%) during oncologic staging or surveillance and 12 (19.35%) during investigations for unrelated conditions. In contrast, 24 patients (38.71%) presented symptomatically, most commonly with pain or functional complaints such as back pain, hip pain, or radicular discomfort (Table 1). This diversity in presentation highlights the importance of including IOH in the differential diagnosis of sclerotic or metabolically active bone lesions. In terms of lesion characteristics, the mean lesion size was 21.8 ± 15.7 mm, ranging from 1.5 to 90 mm.

**Figure 2:**
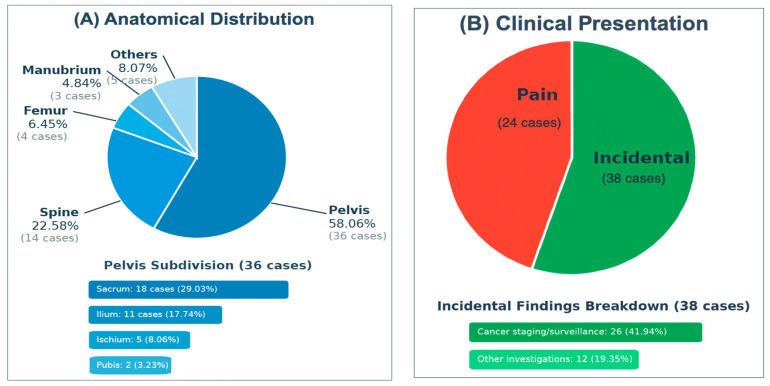
(**A**) The anatomical distribution of IOH, (**B**) The clinical presentation.

Analysis of anatomical distribution demonstrated a marked predilection for the axial skeleton, particularly the pelvic region, which accounted for 36 cases (58.06%). Within the pelvis, the sacrum was the most frequently involved site (18 cases; 29.03%), followed by the ilium (17.74%), ischium (8.06%), and pubis (3.23%). The spine was the second most commonly affected region (14 cases; 22.58%), with thoracic vertebrae involved in 8 cases (12.90%) and lumbar vertebrae in 6 cases (9.68%). Less commonly involved sites included the femur (6.45%), manubrium sterni (4.84%), humerus (3.23%), ribs (3.23%), and tibia (1.61%). The anatomical distribution and clinical presentation patterns are illustrated in Figure 2. A comprehensive summary of the characteristics of the included studies, including study design, sample size, geographical origin, tumor location, and reasons for investigation, is presented in Table 2.

Management approaches were predominantly conservative, with biopsy alone employed in 54 cases (87%). Surgical excision or curettage was performed in 5 cases (8%), while percutaneous thermal ablation, including radiofrequency ablation (RFA) and microwave ablation (MWA) was utilized in 3 cases (5%).

### 3.2. Radiological Features and Imaging Patterns

Table 3 summarizes the characteristic imaging findings of IOH across multiple modalities. On CT imaging, the majority of lesions demonstrated a sclerotic appearance (69.35%), while fewer were lytic (9.68%), mixed (6.45%), or occult (4.84%). In a minority of cases, CT features were not reported (9.68%).

MRI findings (*n* = 46) showed that most lesions were hypointense to isointense on T1-weighted imaging (95.65%), with a heterogeneous signal pattern commonly described. On T2-weighted and short tau inversion recovery (STIR) sequences, the majority were hyperintense (93.48%). Post-contrast enhancement was typically mild to moderate, though not consistently reported.

**Figure 3:**
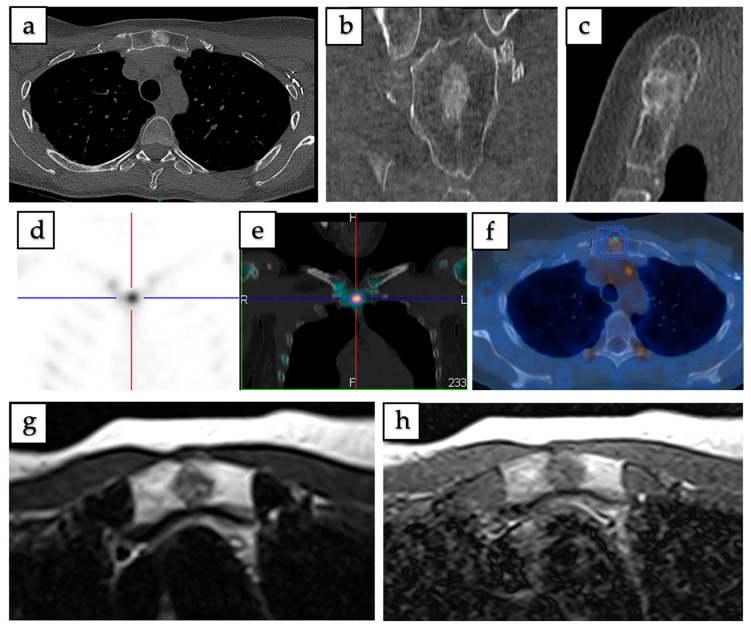
Imaging of intraosseous hibernoma confirmed by biopsy in a 49-year-old female involving the manubrium sterni. (**a**–**c**) Axial, coronal, and sagittal CT scans demonstrate a well-defined 13 mm sclerotic lesion. (**d**,**e**) SPECT-CT reveals increased radiotracer uptake localized to the lesion. (**f**) ^18^F-FDG PET/CT shows mild metabolic uptake with a maximum standardized uptake value (SUVmax = 3.2). (**g**) Axial T1-weighted MRI demonstrates intermediate signal intensity. (**h**) Axial T2-weighted MRI demonstrates a heterogeneous lesion with mild hyperintensity.

**Figure 4:**
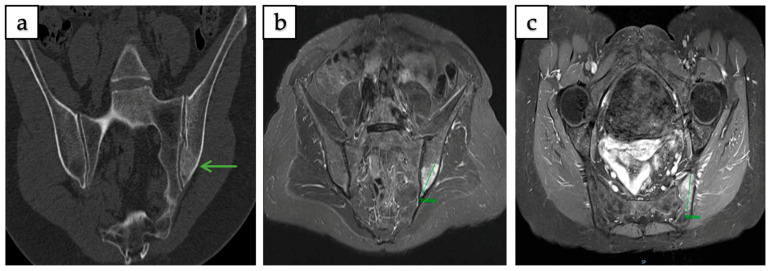
Intraosseous hibernoma of the left iliac bone confirmed by biopsy in a 63-year-old woman, measuring 38 mm in its largest axis. (**a**) Coronal CT scan showing an ill-defined, mildly sclerotic lesion (green arrow). (**b**,**c**) T1-weighted fat-saturated post-gadolinium MRI in coronal and axial planes demonstrating a heterogeneous lesion with contrast enhancement.

^18^F-FDG PET/CT (*n* = 16) revealed increased metabolic activity in 68.75% of cases, with uptake ranging from mild to high. Bone scintigraphy/SPECT (*n* = 18) demonstrated increased tracer uptake in 72.22%, with limited cases showing no uptake. ^68^Gallium-DOTATATE PET/CT (^68^Ga-DOTATATE PET/CT) (*n* = 4) yielded variable results, with equal distribution among no uptake, mild, moderate, and high uptake categories (25% each), reflecting heterogeneous somatostatin receptor expression. Representative examples of CT, MRI, and PET/CT findings of intraosseous hibernoma are shown in Figure 3 and Figure 4.

### 3.3. Comparative Radiological Features of IOH and Metastatic Lesions

Given the frequent diagnostic confusion between intraosseous hibernoma and metastatic bone lesions, a structured comparison of their key radiological features is presented in Table 4. This overview highlights both shared characteristics and distinguishing findings across CT, MRI, ^18^F-FDG PET/CT, and scintigraphy/SPECT.

### 3.4. Immunohistochemical Features

Immunohistochemical profiling showed consistent positivity for S100 (83.87%), with very limited testing and positivity for adipophilin (11.29%) and FABP4/AP2 (1.61%). CD68 showed negativity in 51.61% of tested cases and positivity in 3.23%. Other markers including CD45, CD163, cytokeratin AE1/AE3, Brachyury, HMB45, MDM2, and Melan A were consistently negative when tested. The profiling features are demonstrated in Table 5.

### 3.5. Multi-Dimensional Analysis of Clinical-Radiological Associations

Table 6 presents a multi-dimensional analysis of clinical, radiological, and demographic associations in IOH. A clear female predominance was observed in the overall population (64.5%, 95% CI: 52.1–75.3%), with a prevalence ratio of 1.82 and substantial representation. While subgroup stratification for axial and appendicular distributions was limited by data availability, the female trend remained more pronounced in the appendicular subset. Male representation accounted for 35.5% of cases, with a lower prevalence ratio (0.55) and moderate representation.

Anatomically, lesions overwhelmingly involved the axial skeleton (80.6%, 95% CI: 69.1–88.6%), particularly the pelvis (58.1%) and spine (22.6%), with relative prevalence ratios of 1.39 and 0.29, respectively. In contrast, appendicular skeleton involvement was limited to 19.4%, with minimal representation.

**Figure 5:**
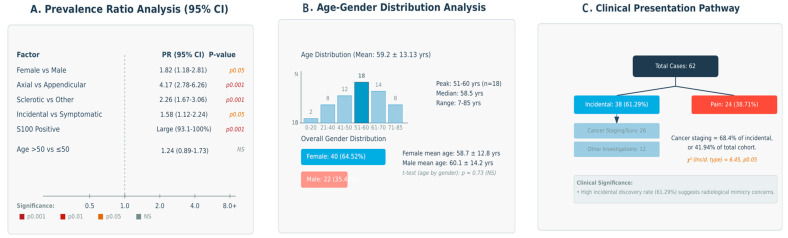
(**A**) The prevalence ratio analysis with confidence intervals, (**B**) age and gender distribution analysis, (**C**) The clinical presentation pathway.

Radiologically, the most frequent manifestation was sclerotic appearance (69.4%, 95% CI: 57.0–79.4%), followed by mixed (21.0%) and lytic patterns (9.7%), corresponding to predominant, moderate, and minimal diagnostic prominence, respectively. Clinically, most lesions were discovered incidentally (61.3%), while symptomatic cases with pain comprised 38.7%, reflecting a dual diagnostic pathway. Immunohistochemically, S100 positivity was universal (52/52 cases), reinforcing its diagnostic reliability with predominant representation. The prevalence ratio analysis with confidence intervals is presented in Figure 5A, while the age-gender distribution analysis is detailed in Figure 5B.

### 3.6. Multiparameter Cross-Correlation

Table 7 presents the multi-parameter cross-correlation matrix of IOH features. Strong correlations (≥0.7) were observed between anatomical distribution and radiological pattern (r = 0.78), and between anatomical distribution and clinical presentation (r = 0.73). Moderate correlations (0.4–0.69) were identified between anatomical distribution and gender predilection (r = 0.56), radiological pattern and clinical presentation (r = 0.54), gender predilection and clinical presentation (r = 0.52), and radiological pattern and gender predilection (r = 0.47).

These correlations suggest possibly relevant relationships, with anatomical distribution showing the strongest associations with both radiological appearance and clinical presentation patterns. The predominant axial skeleton involvement correlates strongly with sclerotic radiological patterns and incidental discovery, while the female predilection shows moderate correlation with specific anatomical and radiological characteristics. The clinical presentation pathway is illustrated in Figure 5C.

### 3.7. Risk of Bias Assessment

The risk of bias assessment of the included studies was conducted using the Murad et al. [16] method specifically designed for case reports and case series. Among the 30 studies assessed, 18 (60%) demonstrated low risk of bias, while 12 (40%) had moderate risk of bias. No studies were classified as having high risk of bias. All studies adequately addressed the Selection domain criteria (patient representativeness and diagnostic accuracy), and most studies provided sufficient details in the Reporting domain to allow for replication of findings or clinical application. The Ascertainment domain (adequacy of exposure and outcome assessment) was generally well-addressed, with all studies meeting these criteria. The Causality domain had the most limitations across studies, with 15 studies (50%) having unclear ratings for “alternative causes ruled out” and 14 studies (46.7%) having unclear ratings for “adequate follow-up duration”. These limitations were mostly observed in studies published before 2015, which generally demonstrated less precise reporting of differential diagnoses and shorter or unspecified follow-up protocols.

## 4. Discussion

IOH represents a rare benign adipocytic neoplasm, characterized by the presence of brown adipose tissue with multivacuolated lipid-rich cells. It was first described by Thorn et al. in 2008, with additional cases gradually emerging in the literature since then [7]. This entity presents significant diagnostic challenges due to its rarity, variable radiological appearance, and mimicry of more aggressive bone lesions, such as metastatic disease [8,10,11].

The pathogenesis of IOH is believed to involve vestigial remnants of brown adipose tissue within the bone marrow [7,38]. These lesions are thought to originate from mesenchymal stem cells, sharing a common lineage with osteoblasts and adipocytes. Molecular pathways involving PRDM16 and BMP7, which are known to regulate brown fat differentiation and bone remodeling, may play a role in the development of IOH [10,39,40]. These factors may also explain the frequent coexistence of sclerotic bone changes and brown fat features in these tumors.

Our systematic review has included a total of 62 cases of histopathologically confirmed IOH reported in the literature, representing the largest and most comprehensive analysis of this rare entity to date. IOH predominantly affects older adults and exhibits a clear female predominance. Clinically, it is most often identified as an incidental finding, frequently discovered during cancer staging or surveillance imaging. However, a significant subset of patients presents with localized, often chronic pain, particularly when the lesion involves weight-bearing or neuroanatomically sensitive regions. This dual presentation, incidental versus symptomatic, reflects both the indolent nature of the disease and its potential to mimic more serious pathology. The correlation between symptoms and anatomical location showed interesting highlights. The pelvis and spine were not only the most common locations but also frequently associated with both incidental discovery and symptomatic presentation. This distribution pattern likely reflects both the routine imaging of the axial skeleton during cancer surveillance and the functional impact of lesions in weight-bearing structures. The anatomical distribution demonstrated a predilection for the axial skeleton, most commonly at the pelvis (58.06%) and spine (22.58%), followed by appendicular locations such as the femur (6.45%).

Radiologically, IOH often resemble both benign and malignant bone lesions, which can complicate diagnosis. On CT, they most commonly appear as solitary, well-defined sclerotic lesions (69.35%) with preserved trabecular architecture. However, lytic (9.68%), mixed (6.45%) or even occult (4.84%) patterns are occasionally reported [6,13,22,26,29]. MRI findings typically include low to intermediate signal intensity on T1-weighted sequences and high signal intensity on T2-weighted or STIR images, often with mild or heterogeneous peripheral enhancement [8,11].

On ^18^F-FDG PET/CT, IOHs display mild to moderate hypermetabolism due to their mitochondrial-rich brown fat cells. ^18^F-FDG PET/CT was performed in 18 cases, revealed mild to moderate hypermetabolism in 72.22% of cases [8,11]. Bone scintigraphy may show mild or moderate radiotracer uptake but is not always diagnostic [8,38].

The limited experience with ^68^Ga-DOTATATE PET/CT, which was included in only a few cases, showed variable uptake patterns ranging from no uptake to high uptake. While this sample is too small for definitive conclusions, it suggests a possible utility in differentiation from other bone lesions, and also warrants further investigation, as ^68^Ga-DOTATATE PET/CT could provide an additional tool for non-invasive differentiation from metastatic disease [37,41].

Given these overlapping imaging features, IOH is frequently misinterpreted as metastatic disease, primary malignant bone tumors such as osteosarcoma, lymphoma, or chordoma, or benign entities including intraosseous lipoma, fibrous dysplasia, hemangioma, and benign notochordal cell tumors [6,8]. This radiologic ambiguity underscores the importance of obtaining histopathological confirmation, particularly in patients with a history of malignancy.

Biopsy remains essential for definitive diagnosis of IOH. Histologically, IOH is characterized by sheets or clusters of multivacuolated adipocytes resembling brown fat cells, with foamy cytoplasm, small central nuclei, and minimal atypia. A distinguishing feature is the preservation of bony trabeculae, which are infiltrated rather than destroyed, contrasting with intraosseous lipoma, which often erodes trabeculae [8,13,24]. Reactive sclerosis of adjacent bone may be seen. The histological appearance can mimic foamy histiocytosis, liposarcoma, or even lipid-laden macrophages, making immunohistochemical profiling essential. IOH cells are positive for S-100 protein, confirming their adipocytic, specifically brown fat, origin. They are negative for CD68, pan-cytokeratin, and desmin, thereby excluding histiocytic, epithelial, and muscle-derived tumors. Importantly, additional tumor markers, including cytokeratin (AE1/AE3) and epithelial membrane antigen (EMA), may be used to exclude metastatic carcinoma. In cases where chordoma is in the differential, especially in axial skeletal lesions, brachyury immunostaining is a valuable marker. IOH is negative for brachyury, whereas chordomas are typically strongly positive, aiding in their distinction.

While there are no pathognomonic genetic mutations reported for IOH. Our review identified only one study that investigated chromosome 11q status in IOH [22]. In this case report, a rearrangement involving chromosome 11q13 was observed, mirroring the genetic alteration frequently found in soft tissue hibernomas and suggesting a possible shared molecular pathway [42,43,44]. Although based on limited data, this finding raises the possibility that 11q abnormalities could serve as a diagnostic marker for IOH, particularly in diagnostically challenging cases. However, further molecular studies are necessary to validate this potential marker and enhance diagnostic accuracy.

A major clinical challenge lies in differentiating IOH from metastatic bone lesions. The metastatic mimicry of IOH represents a diagnostic pitfall with possible risk for serious consequences. Though both can appear sclerotic, lytic, or mixed, IOH typically exhibits well-circumscribed sclerotic lesions on CT with trabecular thickening and no cortical destruction or soft tissue extension. In contrast, metastases often show more aggressive features, including cortical breach and soft tissue masses [8,11]. MRI can further aid in differentiation. IOH demonstrates moderate, often peripheral enhancement, whereas metastases tend to show intense and diffuse contrast enhancement [17,24]. Additionally, FDG uptake in IOH is generally mild to moderate, in contrast to the marked hypermetabolism seen in metastatic disease [6,11,17,19]. Nonetheless, due to the substantial imaging overlap, biopsy remains the gold standard for definitive diagnosis, particularly in oncologic patients or when imaging features are ambiguous. In our opinion, the most reliable differentiating features include characteristic MRI signal patterns and S100 positivity on histopathological examination.

As demonstrated in our multi-dimensional analysis, there were significant associations between gender, anatomical location, and radiological features. Sclerotic lesions were significantly more common in the axial skeleton, especially at the pelvis, and were associated with incidental discovery patterns. The axial skeleton predominance may reflect both the predilection for brown adipose tissue distribution and the increased likelihood of detection during routine cross-sectional imaging.

Our analysis identified several gender-based differences in tumor location and presentation. Female patients demonstrated significantly higher odds of overall involvement and showed correlation with the predominant axial skeleton distribution pattern. This gender-based anatomical distribution has not been previously recognized in the literature and may reflect underlying biological differences in brown adipose tissue metabolism or distribution between genders.

In most cases, IOH requires no treatment beyond diagnosis and observation, particularly when lesions are asymptomatic and discovered incidentally. However, symptomatic cases—especially those involving weight-bearing bones or neural structures—may present with persistent pain. In such scenarios, conservative management is generally sufficient, though minimally invasive interventions, such as RFA MWA, have been reported to provide effective symptom relief with low morbidity [9,26,36]. Surgical excision or curettage has also been performed in selected cases, particularly when diagnostic uncertainty persists or structural risk is present [17,21,25,31,35]. Importantly, IOH is a benign, non-metastasizing tumor with no reported cases of malignant transformation, and its prognosis is uniformly favorable once appropriately diagnosed.

This study is subject to several limitations inherent to literature-based reviews. The majority of available evidence consists of single-patient case reports and a small number of case series. Such evidence is prone to publication and reporting bias, and heterogeneity in data quality may affect the generalizability of the findings. In addition, incomplete documentation of clinical, radiological, and pathological details in some cases restricted the ability to perform subgroup analyses or draw definitive correlations. The retrospective nature of the data also precluded uniform imaging interpretation or histopathologic reassessment. Furthermore, genetic and molecular data were sparsely reported, limiting insight into the underlying pathogenesis and potential diagnostic biomarkers. Future prospective studies, multicenter collaborations, and the establishment of rare tumor registries will be essential to strengthen the evidence base and improve recognition of IOH in clinical practice.

## 5. Conclusions

Intraosseous hibernoma is a rare, benign bone tumor that often mimics more aggressive lesions on imaging. While typically asymptomatic and found incidentally, some cases present with pain, particularly in the appendicular skeleton. Diagnosis relies on biopsy and S100-positive histopathology to exclude malignancy. Most cases require no treatment, though symptomatic lesions may benefit from minimally invasive intervention. Awareness of this entity is essential to avoid misdiagnosis and overtreatment.

## Figures and Tables

**Table 1:** Demographics and Characteristics of Intraosseous Hibernoma Cases.

Characteristic	Value
**Patient Demographics:**	
Age, years, mean ± SD (range)	59.2 ± 13.13 (7–85)
Sex	
Male, *n* (%)	22 (35.48%)
Female, *n* (%)	40 (64.52%)
**Clinical Presentation:**	
Incidental findings, n (%)	38 (61.29%)
*- During cancer staging/surveillance*	26 (41.94%)
*- During other medical investigations*	12 (19.35%)
Pain, n (%)	24 (38.71%)
**Lesion Characteristics:**	
Size in mm, mean ± SD (range)	21.8 ± 15.7 (1.5–90)
**Anatomical Location:**	
Pelvis, total:	36 (58.06%)
*- Sacrum*	18 (29.03%)
*- Ilium*	11 (17.74%)
- Ischium	5 (8.06%)
*- Pubis*	2 (3.23%)
Spine, total:	14 (22.58%)
*- Thoracic*	8 (12.90%)
*- Lumbar*	6 (9.68%)
Femur	4 (6.45%)
Manubrium sterni	3 (4.84%)
Humerus	2 (3.23%)
Ribs	2 (3.23%)
Tibia	1 (1.61%)
**Management**	
Conservative (biopsy only)	54 (87%)
Surgical excision/curettage	5 (8%)
percutaneous thermal ablation (RFA, MWA)	3 (5%)

RFA: Radiofrequency ablation, MWA: Microwave ablation, SD: Standard deviation.

**Table 2:** Detailed Characteristics of Included Studies on Intraosseous Hibernoma.

Author, Year	Country	Study Design	Sample Size	Tumor Location	Investigation Reason	Imaging Modality Used
Shaikh et al., 2025 [34]	USA	Case Report	1	Left iliac bone (pelvis)	Found incidentally during investigation of fall injury	CT, MRI
Tonkaz et al., 2024 [33]	Turkey	Case Report	1	Femur (greater trochanter)	Discovered incidentally after a fall	CT, MRI, Bone scintigraphy/SPECT
Gangahar et al., 2023 [37]	USA	Case Series	18	Multiple locations: femur (7), pelvis (4), spine (3), humerus (2), tibia (1), ribs (1)	Various (surveillance, back pain, staging, limping, weight loss, sciatica)	CT, MRI, ^18^F-FDG PET/CT, Bone scintigraphy/SPECT, ^68^Ga-DOTATATE PET/CT
Samargandi et al., 2023 [6]	France	Case Report	2	Manubrium sterni, left iliac bone	Incidentally found during breast cancer staging, incidental bony lesion on MRI	CT, MRI, ^18^F-FDG PET/CT, Bone scintigraphy/SPECT
Song M et al., 2023 [31]	China	Case Report	1	Left seventh rib	Paroxysmal left-sided thoracic pain for 2 months	CT
Stolte et al., 2023 [11]	Switzerland	Case Report	1	Upper left pubic bone (pelvis)	Lesion discovered incidentally on ^18^F-FDG PET/CT during lung cancer staging	CT, ^18^F-FDG PET/CT
Gitto et al., 2022 [35]	Netherlands	Case Report	1	Right proximal humerus	Shoulder MRI incidentally revealed an osseous lesion	MRI
Srinivasan et al., 2022 [30]	India	Case Report	1	Left proximal tibia	Painless solitary mass, slowly growing over 2 years	MRI, ^18^F-FDG PET/CT
Weiss et al., 2022 [12]	USA	Case Report	2	Right sacral wing, right sacral ala	Chronic right hip pain, chronic midline lumbar back pain	CT, MRI, ^18^F-FDG PET/CT
Ko et al., 2020 [9]	USA	Case Report	1	T3 vertebral body (spine)	Incidental finding during ovarian cancer workup	CT, MRI
Mi-Kyung Um et al., 2020 [32]	South Korea	Case Report	1	T7 vertebral body (spine)	Discovered incidentally on chest CT during tuberculosis screening	CT, MRI, ^18^F-FDG PET/CT
Faropoulos et al., 2019 [25]	Greece	Case Report	1	Left sacral vertebra S4 (spine)	Low back pain extending to the left buttock, causalgia	CT, MRI
Myslicki et al., 2019 [13]	USA	Case Series	5	L2 vertebral body, right sacral ala, Right hemisacrum at S2, left posterior superior iliac spine, left proximal femur	Various (incidental findings, pelvic pain)	CT, MRI, Bone scintigraphy/SPECT
Woodford et al., 2019 [29]	Australia	Case Report	1	Right sacral ala (pelvis)	Identified on ^68^Ga-DOTATATE PET/CT during neuroendocrine tumor staging	CT, ^18^F-FDG PET/CT, ^68^Ga-DOTATATE PET/CT
Song B et al., 2017 [8]	South Korea	Case Series	6	L3 vertebral body, T12 vertebral body (2), sacral ala, distal femur, L3–4 vertebral body	Five patients with musculoskeletal pain, one incidental finding	CT, MRI, ^18^F-FDG PET/CT, Bone scintigraphy/SPECT
Zuidberg-van der Gronde et al., 2017 [28]	Netherlands	Case Report	1	Manubrium sterni	Found during annual MRI breast cancer follow-up	CT, MRI, Bone scintigraphy/SPECT
Dannheim et al., 2016 [27]	USA	Case Report	1	Right sacral ala (pelvis)	Incidental finding during staging of breast cancer work-up	CT, ^18^F-FDG PET/CT
Yahia et al., 2016 [21]	Tunisia	Case Report	1	Right fourth rib	Right chest pain for 6 months	CT, Bone scintigraphy/SPECT
Vlychou et al., 2016 [17]	United Kingdom	Case Report	1	Left ischium (pelvis)	Left sacral/hip pain radiating down the left leg, worse at night	CT, MRI, ^18^F-FDG PET/CT
Westacott et al., 2016 [20]	Australia	Case Report	1	Sacrum (pelvis)	Right hip and lower back pain	CT
Degnan et al., 2015 [26]	USA	Case Report	1	Right S2 sacral lesion (spine)	Persistent lower back and right buttock pain for months	CT, MRI
Hafeez et al., 2015 [18]	USA	Case Report	1	L3 vertebral body (spine)	No pain reported; identified incidentally during imaging	CT, MRI
Jerman et al., 2015 [19]	Slovenia	Case Report	1	Left sacrum (pelvis)	Chronic lower back pain (lumbago, worse after physical activity)	CT, MRI, ^18^F-FDG PET/CT, Bone scintigraphy/SPECT
Bonar et al., 2014 [24]	Australia	Case Series	5	T5 vertebral body, Manubrium sterni, left ischiopubic ramus, T12 vertebral body, left iliac crest	All incidental findings during staging investigations	CT, MRI, ^18^F-FDG PET/CT, Bone scintigraphy/SPECT
Bai et al., 2013 [10]	USA	Case Report	1	Right ilium (pelvis)	Posterolateral right hip pain worsened by activity	CT, MRI, ^18^F-FDG PET/CT
Botchu et al., 2013 [22]	United Kingdom	Case Report	1	Right posterior ilium (pelvis)	Low back and buttock pain for six months	CT, MRI, Bone scintigraphy/SPECT
Lynch et al., 2013 [38]	USA	Case Report	1	Left iliac crest (pelvis)	Incidental finding as part of workup for essential thrombocytopenia	NR *
Ringe et al., 2013 [36]	Germany	Case Report	1	Left sacral bone (pelvis)	Lower back pain radiating to the left foot, especially at night; therapy-refractory	CT, MRI
Kumar et al., 2011 [23]	USA	Case Report	1	Left sacral ala (pelvis)	Low back pain radiating to the left lower limb	CT, MRI
Thorns et al., 2008 [7]	Germany	Case Report	1	Ilium (pelvis)	Incidental finding during workup	NR *

* NR: Not reported; CT: Computed tomography; MRI: Magnetic resonance imaging; ^18^F-FDG PET/CT: ^18^F-fluorodeoxyglucose positron emission tomography/computed tomography; SPECT: Single-photon emission computed tomography; ^68^Ga-DOTATATE PET/CT: Gallium-68 DOTA-TATE positron emission tomography/computed tomography.

**Table 3:** Multi-modality Imaging Pattern Recognition.

Imaging Modality and Characteristics	Number (%)
**CT Findings: (n = 62)**	
Appearance pattern	
- Sclerotic	43 (69.35%)
- Lytic	6 (9.68%)
- Mixed (sclerotic + lytic)	4 (6.45%)
- Occult	3 (4.84%)
- Not reported	6 (9.68%)
**MRI Characteristics:(n = 46)**	
T1-weighted signal	
- Hypointense/low—Isointense	44 (95.65%)
- Hyperintense/high	2 (4.35%)
- Heterogeneous	Common finding
T2-weighted signal/STIR	
- Hyperintense/high	43 (93.48%)
- Hypointense/low—Isointense	3 (6.52%)
Post-contrast enhancement	
- Present (mild to moderate)	Majority of reported cases
- Absent	Minority of reported cases
**^18^F-FDG PET/CT Findings (n = 16):**	
*Mean SUV (3.31 ± 0.67)*	
- No uptake	4 (25%)
- Uptake	11 (68.75%)
Mild	9
Moderate	1
High	1
- Not reported	1 (6.25%)
**Bone scintigraphy/SPECT Findings (n = 18):**	
- Increased uptake	13 (72.22%)
- No uptake	2 (11.11%)
- Not reported	3 (16.67%)
**^68^Ga-DOTATATE PET/CT Findings (n = 4):**	
- No uptake	1 (25%)
- Mild uptake	1 (25%)
- Moderate uptake	1 (25%)
- High uptake	1 (25%)

CT: Computed tomography; MRI: Magnetic resonance imaging; STIR: Short tau inversion recovery; ^18^F-FDG PET/CT: ^18^F-fluorodeoxyglucose positron emission tomography/computed tomography; SUV: Standardized uptake value; SPECT: Single-photon emission computed tomography; ^68^Ga-DOTATATE PET/CT: Gallium-68 DOTA-TATE positron emission tomography/computed tomography.

**Table 4:** Key Radiological Features for Differentiating Intraosseous Hibernoma from Metastatic Lesions.

Feature	Intraosseous Hibernoma	Metastatic Lesion
Number	Almost always solitary	Solitary or multiple
Margins	Well-defined	Less defined, irregular
Density on CT	Sclerotic, sometimes mixed/lytic and rarely occult	Sclerotic, lytic, or mixed
MRI Signal (T1/T2)	Low/intermediate T1; hyperintense T2	Low T1; variable T2
Contrast Enhancement	Heterogeneous, low to moderate, peripheral possible	Diffuse, intense
^18^F-FDG PET/CT	Mild or moderate uptake, SUV usually low-to-intermediate	Markedly hypermetabolic, SUV often high
Bone Scintigraphy/SPECT	Mild uptake, may be variable	Typically, increased uptake
Preservation of Trabeculae	Common	Often destroyed/disrupted
Cortical Breach/Soft Tissue	Rare	Possible, especially aggressive tumors
**Typical Location**	Common in axial skeleton	Any bone

CT: Computed tomography; MRI: Magnetic resonance imaging; ^18^F-FDG PET/CT: ^18^F-fluorodeoxyglucose positron emission tomography; SUV: Standardized uptake value; SPECT: Single-photon emission computed tomography.

**Table 5:** Immunohistochemical Profile of Intraosseous Hibernoma.

Marker	Positive *n* (%)	Negative *n* (%)	Not Reported *n* (%)
S100	52 (83.87%)	0	10 (16.13%)
Adipophilin	7 (11.29%)	0	55 (88.71%)
FABP4/AP2	1 (1.61%)	0	61 (98.39%)
CD68	1 (1.61%)	32 (51.61%)	29 (46.77%)
CD45	0	12 (19.35%)	50 (80.65%)
CD163	0	14 (22.58%)	48 (77.42%)
Cytokeratin AE1/AE3	0	42 (67.74%)	20 (32.26%)
Brachyury	0	14 (22.58%)	48 (77.42%)
HMB45	0	9 (14.52%)	53 (85.48%)
MDM2	0	2 (3.23%)	60 (96.77%)
Melan A	0	7 (11.29%)	55 (88.71%)

FABP4/AP2: Fatty acid–binding protein 4/Adipocyte protein 2; CD68: Cluster of differentiation 68; CD45: Cluster of differentiation 45; CD163: Cluster of differentiation 163; cytokeratin AE1/AE3: Broad-spectrum cytokeratin cocktail AE1/AE3; HMB45: Human melanoma black-45; MDM2: Mouse double minute 2 homolog; Melan A: Melanoma antigen recognized by T cells; S100: S100 calcium-binding protein.

**Table 6:** Multi-Dimensional Analysis of Clinical-Radiological Associations in Intraosseous Hibernoma.

Parameter	Stratification	Observed Frequency	Proportion (95% CI) ^1^	Prevalence Ratio ^2^	Estimated Representation ^3^
**Gender-Based Distribution:**					
Female preponderance	Overall population	40/62	64.5% (52.1–75.3%)	1.82	+++
	Axial distribution subset	^4^	^4^	^4^	++
	Appendicular distribution subset	^4^	^4^	^4^	+++
Male representation	Overall population	22/62	35.5% (24.7–47.9%)	0.55	++
**Anatomical Distribution Analysis:**					
Axial skeleton involvement	Aggregate analysis	50/62	80.6% (69.1–88.6%)	4.17	++++
- Pelvis	Subgroup analysis	36/62	58.1% (45.7–69.5%)	1.39	+++
- Spine	Subgroup analysis	14/62	22.6% (13.8–34.8%)	0.29	++
Appendicular skeleton	Comparative analysis	12/62	19.4% (11.4–30.9%)	0.24	+
**Radiological Manifestation Patterns:**					
Sclerotic presentation	Primary pattern	43/62	69.4% (57.0–79.4%)	2.26	++++
Lytic presentation	Alternative pattern	6/62	9.7% (4.5–19.5%)	0.11	+
Mixed/Other presentations	Variant patterns	13/62	21.0% (12.5–33.0%)	0.26	++
**Clinical-Pathological Correlation:**					
Incidental discovery	Diagnostic pathway analysis	38/62	61.3% (48.9–72.4%)	1.58	+++
Pain manifestation	Symptomatic presentation	24/62	38.7% (27.6–51.1%)	0.63	++
**Immunohistochemical Profile Stratification:**					
S100 positivity	Diagnostic marker efficacy	52/52	100% (93.1–100%) ^5^	∞	++++

Notes: ^1^ Wilson score method confidence intervals ^2^ Calculated as observed proportion/(1-observed proportion) ^3^ Qualitative assessment scale: + minimal, ++ moderate, +++ substantial, ++++ predominant ^4^ Subgroup analysis not possible with aggregate data ^5^ One-sided 97.5% confidence interval used for 100% proportion. ∞: Ratio not defined due to 100% prevalence

**Table 7:** Multi-Parameter Cross-Correlation Matrix of Intraosseous Hibernoma Features.

Variable	Anatomical Distribution	Radiological Pattern	Gender Predilection	Age Distribution	Clinical Presentation	Immunological Profile
Anatomical Distribution	1.00	0.78 **	0.56 *	0.22	0.73 **	0.18
Radiological Pattern	0.78 **	1.00	0.47 *	0.19	0.54 *	0.21
Gender Predilection	0.56 *	0.47 *	1.00	0.25	0.52 *	0.15
Age Distribution	0.22	0.19	0.25	1.00	0.27	0.14
Clinical Presentation	0.73 **	0.54 *	0.52 *	0.27	1.00	0.23
Immunological Profile	0.18	0.21	0.15	0.14	0.23	1.00

Notes: Strong (≥0.7) and moderate (0.4–0.69) patterns suggest potential clinically relevant relationships, while significance indicators (* and **) estimate the probability that observed distributions exceed random chance.
